# Multimerization of the GATA4 transcription factor regulates transcriptional activity and cardiomyocyte hypertrophic response

**DOI:** 10.7150/ijbs.65664

**Published:** 2022-01-01

**Authors:** Satoshi Shimizu, Yoichi Sunagawa, Naruto Hajika, Natsumi Yorimitsu, Yasufumi Katanasaka, Masafumi Funamoto, Yusuke Miyazaki, Nurmila Sari, Kana Shimizu, Koji Hasegawa, Tatsuya Morimoto

**Affiliations:** 1 Division of Molecular Medicine, School of Pharmaceutical Sciences, University of Shizuoka, Shizuoka, Japan; 2 Division of Translational Research, Clinical Research Institute, National Hospital Organization Kyoto Medical Center, Kyoto, Japan; 3 Shizuoka General Hospital, Shizuoka, Japan

**Keywords:** GATA4, Multimerization, Acetylation, Cardiomyocyte, Hypertrophy

## Abstract

The activation of the GATA-binding factor 4 (GATA4) transcription factor induces cardiac hypertrophy and heart failure. The multimerization of transcription factors often plays an important role in the regulation of transcriptional activity. Here, we report that the GATA4 transcription factor forms a homomultimer and that residues 308-326 of GATA4 are necessary for its multimerization. The acetylation of GATA4 by the transcriptional co-activator p300 induces the multimerization of GATA4 and activates its DNA binding activity. In addition, we found that the suppression of GATA4 multimerization did not reduce its acetylation, but repressed GATA4/p300-induced gene transcription. Furthermore, the inhibition of GATA4 multimerization suppressed phenylephrine (PE)-induced hypertrophic response in cardiomyocytes. This study demonstrates that the multimerization of GATA4 during the p300-induced acetylation of GATA4 activates the transcription of hypertrophic response genes, which leads to cardiomyocyte hypertrophy. Therefore, the inhibition of GATA4 homomultimerization could serve as a potential therapeutic strategy for the development of novel drugs against heart failure.

## Introduction

The number of individuals with heart failure is estimated to be 26 million worldwide in 2014, making it a major contributor to global deaths 1, 2. As the current 5-year survival rate for heart failure is less than 60% 3, 4, it is vitally important to develop novel treatment strategies for this disease. Previous studies have demonstrated that a variety of cardiac stress factors primarily activate the renin-angiotensin system and the sympathetic nervous system, which results in cardiomyocyte hypertrophy and the development of heart failure 5. The drugs that are presently used for the treatment of heart failure aim to suppress the functionality of the renin-angiotensin and sympathetic nervous systems. These drugs include angiotensin-converting enzyme inhibitors, angiotensin II receptor blockers, mineralocorticoid receptor antagonists, and beta-adrenergic blockers. These drugs target the upstream proteins of the cardiac hypertrophic response pathways, including cell surface receptors. However, as several other upstream proteins are involved in the development and progression of heart failure, these drugs are incapable of targeting all the hypertrophic signaling cascades, resulting in insufficient therapeutic efficacy 3, 4. The hypertrophic signaling cascades of cardiomyocytes usually converge on a nuclear signaling pathway, resulting in alterations in the gene expression pattern. A thorough investigation of the nuclear pathways involved in the transcription of cardiac hypertrophic response genes is necessary.

GATA4 is a zinc finger transcription factor that is expressed at an early stage of embryonic development and is involved in the formation of the heart 6, 7. We have previously demonstrated that the transcriptional co-activator, p300, localized in the nucleus of cardiomyocytes, induces the acetylation of GATA4 during the development of heart failure. The acetylation of GATA4 increases its DNA binding activity and the transcription of cardiac hypertrophic response genes, including atrial natriuretic factor (ANF), brain natriuretic peptide (BNP), endothelin-1 (ET-1), and β-myosin heavy chain (β-MHC), leading to cardiomyocyte hypertrophy 8, 9, 10. The inhibition of p300-dependent acetylation of GATA4 suppresses the transcription of cardiac hypertrophic response genes, cardiomyocyte hypertrophy, and the development of heart failure, suggesting that regulation of the transcriptional activity of GATA4 could serve as a novel therapeutic strategy for heart failure 11, 12.

It has been reported that GATA4 interacts with various transcription factors, including T-box transcription factor 5 (TBX5), myocyte enhancer factor 2 (MEF2), serum response factor (SRF), and NK2 homeobox 5 (Nkx2.5), as well as transcriptional cofactors, including cyclin-dependent kinase-9 (CDK9), heart- and neural crest derivatives-expressed protein 2 (HAND2), receptor for activated protein kinase C1 (RACK1), and nuclear factor of activated T-cells, cytoplasmic 4 (NFATC4) 13-17. Furthermore, it has been reported that post-translational modifications of GATA4, including acetylation and phosphorylation by p300 and extracellular signal-regulated kinase (ERK1/2), increase its DNA binding and transcriptional activities 18. However, the mechanisms underlying the transcriptional regulation of GATA4 by these GATA4-binding proteins remain unclear.

In general, transcription factors, including tumor protein p53 (p53), signal transducers and activator of transcription 3 (STAT3), and cAMP responsive element binding protein (CREB), regulate transcriptional activity by forming complexes such as homodimers. Estrogen receptors and glucocorticoid receptors, which are zinc finger transcription factors like GATA4, increase transcriptional activity by forming homodimers 19, 20. GATA1, another member of the GATA family, regulates transcriptional activity by forming homodimers 21. Therefore, we hypothesized that GATA4 undergoes homodimerization, which regulates its transcriptional activity. In this study, we investigated the relationship between the dimerization of GATA4 and its transcriptional activation and determined whether GATA4 dimers regulate hypertrophic responses in cardiomyocytes.

## Results

### GATA4 formed a dimer both *in vitro* and within cells

**Figure 1:**
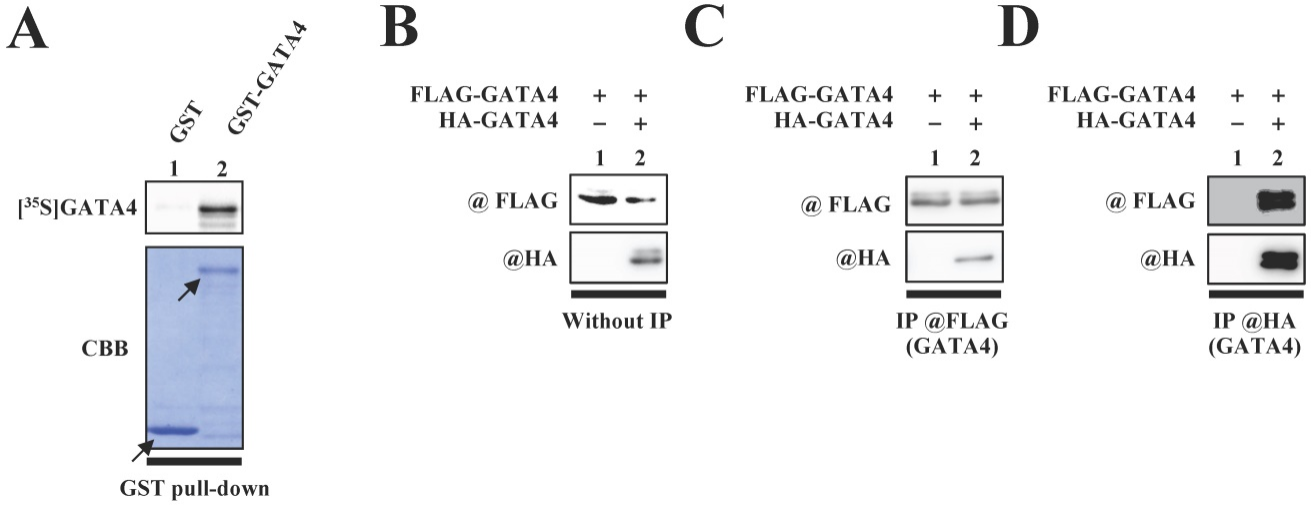
The formation of GATA4 homodimers was observed both *in vitro* and *within cells*. (A) An ^35^S-labelled GATA4 protein was translated *in vitro* and incubated with GST alone or GST-full length GATA4 immobilized on Glutathione-Sepharose 4B. The binding of GST-full length GATA4 to the ^35^S-labeled GATA4 was detected using a BAS2000 bio-imaging analyzer. The arrow indicates GST alone or GST-GATA4. (B, C, D) Nuclear extracts from HEK293T cells expressing FLAG-GATA4 and HA-GATA4 were immunoprecipitated with anti-FLAG or anti-HA antibodies and analyzed by Western blotting with anti-HA and anti-FLAG antibodies.

We first investigated whether GATA4 undergoes homodimerization *in vitro*. A glutathione S-transferase (GST) pull-down assay was performed with an ^35^S-labelled GATA4 protein using GST-GATA4. The results demonstrated that GST-GATA4 bound to the ^35^S-labeled GATA4 (Fig.1A). In order to investigate whether GATA4 formed a homodimer in the nuclear extracts from HEK293T cells, we subsequently performed immunoprecipitation with anti-hemagglutinin (HA) tag or anti-FLAG antibodies, followed by Western blotting. As depicted in Fig. 1C and D, HA-GATA4 bound to FLAG-GATA4. These results suggested that GATA4 forms a homodimer both *in vitro* and within the cells.

### Residues 308-326 of GATA4 were crucial for dimerization

**Figure 2:**
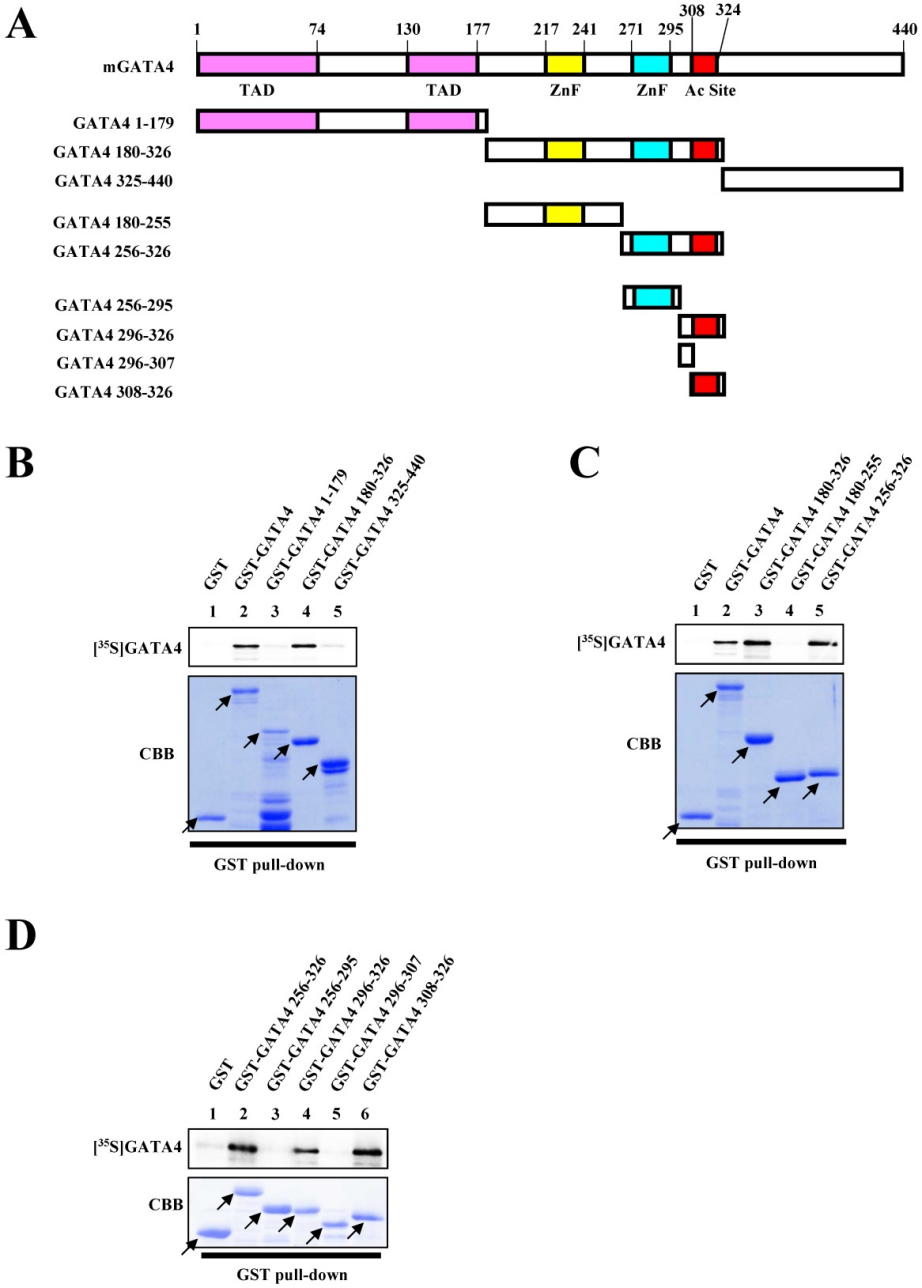
Residues 308-326 of GATA4 bound to the full length GATA4. (A) Schematic representation of the full length GATA4 protein and deletion mutants. (B, C, D) An ^35^S-labelled GATA4 protein, translated *in vitro*, was incubated with GST alone, GST-full length GATA4, or GATA4 mutants immobilized on Glutathione-Sepharose 4B. The binding of GST-full length GATA4 and GATA4 mutants to ^35^S-labeled GATA4 was detected using a BAS2000 bio-imaging analyzer. The arrow indicates GST alone, GST-full length GATA4, or GST-GATA4 deletion mutants. TAD, transcriptional activation domain; ZnF, zinc finger motif; Ac Site, acetylation site.

In order to identify the dimerization region necessary for the homodimerization of GATA4, we performed a series of GST pull-down assays. Various deletion mutants of GATA4 were fused with GST (Fig. 2A). As depicted in Fig. 2B, residues 180-326 of GATA4 bound to the ^35^S-labeled GATA4. More specifically, residues 256-326 of GATA4 bound to the ^35^S-labeled GATA4, while residues 180-255 did not, indicating that residues 256-326 of GATA4 are important for its homodimerization (Fig. 2C). Moreover, three deletion mutants in the 308-326 residue stretch also bound to GATA4 (Fig. 2D). These results indicated that residues 308-326 of GATA4 are crucial for its homodimerization.

### GATA4 homodimerization was increased by p300-induced acetylation

**Figure 3:**
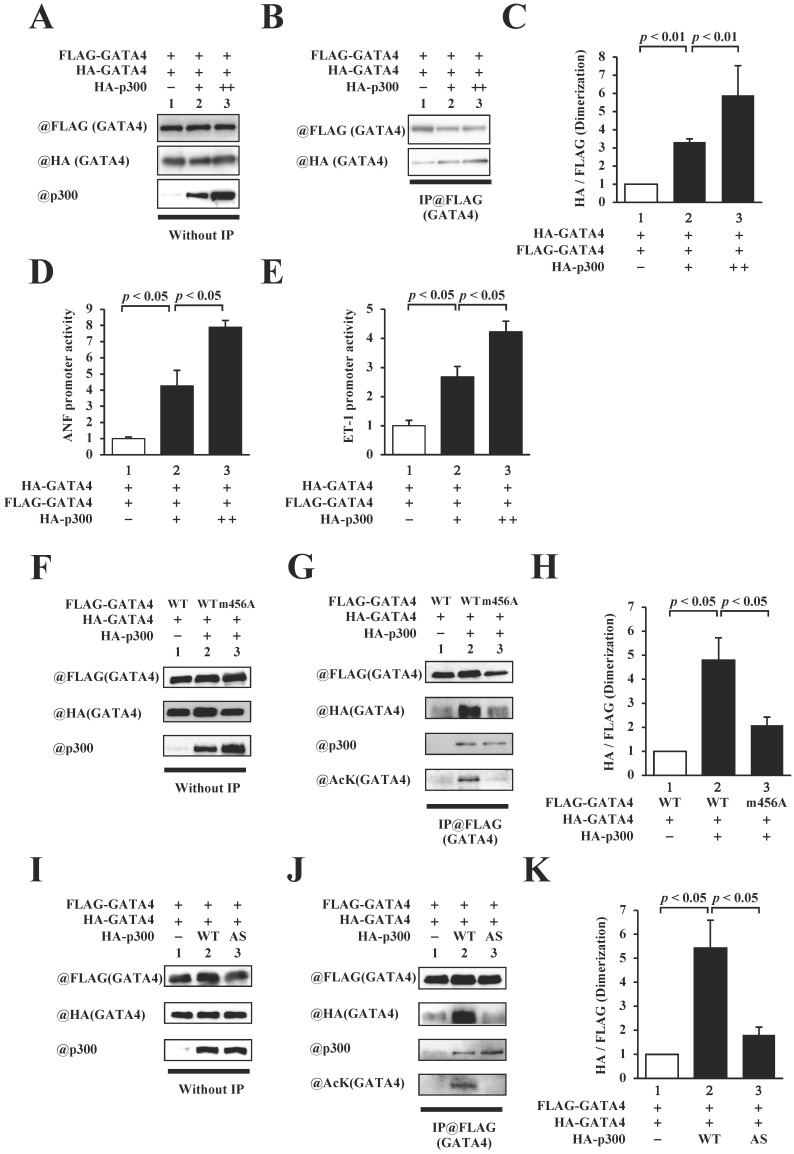
The homodimerization of GATA4 was increased by p300-induced acetylation. (A, B, C) HEK293T cells were transfected with FLAG-GATA4, HA-GATA4, and HA-p300. The nuclear extracts from these cells were immunoprecipitated with ANTI-FLAG^®^ M2 Agarose Affinity Gel and subjected to Western blotting using anti-HA and anti-FLAG antibodies. The signal levels of HA-GATA4 were measured relative to those of FLAG-GATA4. (D, E) The HEK293T cells were co-transfected with pANF-luc (D) or pET-1-luc (E), and with pRL-SV40, FLAG-GATA4, and HA-p300. Relative promoter activity was calculated based on the ratio of firefly to sea pansy luciferase activity. (F, G, H) HEK293T cells were co-transfected with FLAG-GATA4 m456A, which is not acetylated by p300, HA-GATA4, or HA-p300. (I, J, K) The HEK293T cells were co-transfected with FLAG-GATA4, HA-GATA4, and the HA-p300 AS, which is an acetyltransferase activity-deficient mutant. The immunoprecipitated samples using ANTI-FLAG^®^ M2 Agarose Affinity Gel were subjected to Western blotting with the anti-HA, anti-FLAG, anti-p300, and anti-acetyl lysine antibodies. The results are presented as the mean ± S.E. of data obtained from three independent experiments. AcK, acetyl lysine.

As residues 308-326 of GATA4 include lysines that are acetylated by p300, we investigated the relationship between p300 and homodimerization of GATA4. To this end, HEK293T cells were co-transfected with FLAG-GATA4, HA-GATA4, and p300 (Fig. 3A). Immunoprecipitation studies were subsequently performed using an anti-FLAG antibody, followed by Western blotting using an anti-HA antibody. As depicted in Fig. 3B and C, the co-expression of p300 increased homodimerization dose dependently. In order to investigate whether GATA4 dimerization depends on transcriptional activation, reporter gene assays were performed with HEK293T cells using ANF and ET-1 promoter-luciferase constructs. The results showed that p300 enhanced the promoter activity of ANF (Fig. 3D) and ET-1(Fig. 3E) in a dose-dependent manner. These results indicated that the expression level of p300 was associated with increases in GATA4 dimerization and GATA4-dependent promoter activation. The transcriptional coactivator p300 possesses acetyltransferase activity in addition to scaffold and bridge functions 22. In order to elucidate whether the acetyltransferase activity, scaffold function, and bridge function of p300 play crucial roles in the homodimerization of GATA4, we co-transfected HEK293T cells with GATA4 K311A/K318A/K320A/K322A (m456A), which can bind to p300 but is not acetylated, or p300 W1466A/Y1467S (AS), which can bind to GATA4 but does not acetylate it, instead of the wild-type proteins, followed by analyses with immunoprecipitation studies and Western blotting. The results revealed that the acetylation and dimerization of GATA4 m456A were not higher than those of the wild type (Fig. 3D, E). Similarly, when p300AS was used, the acetylation and dimerization of GATA4 did not increase in comparison to those of the wild-type p300 (Fig. 3G, H). It has been previously reported that GATA4 m456A interacts with p300 but does not undergo acetylation 8. It has also been reported that p300 AS interacts with GATA4 but loses its acetyltransferase activity due to this mutation 9, 10. These findings suggest that the acetyltransferase activity of p300 induced the homodimerization of GATA4.

### GATA4 formed a multimer that had at least three subunits

**Figure 4:**
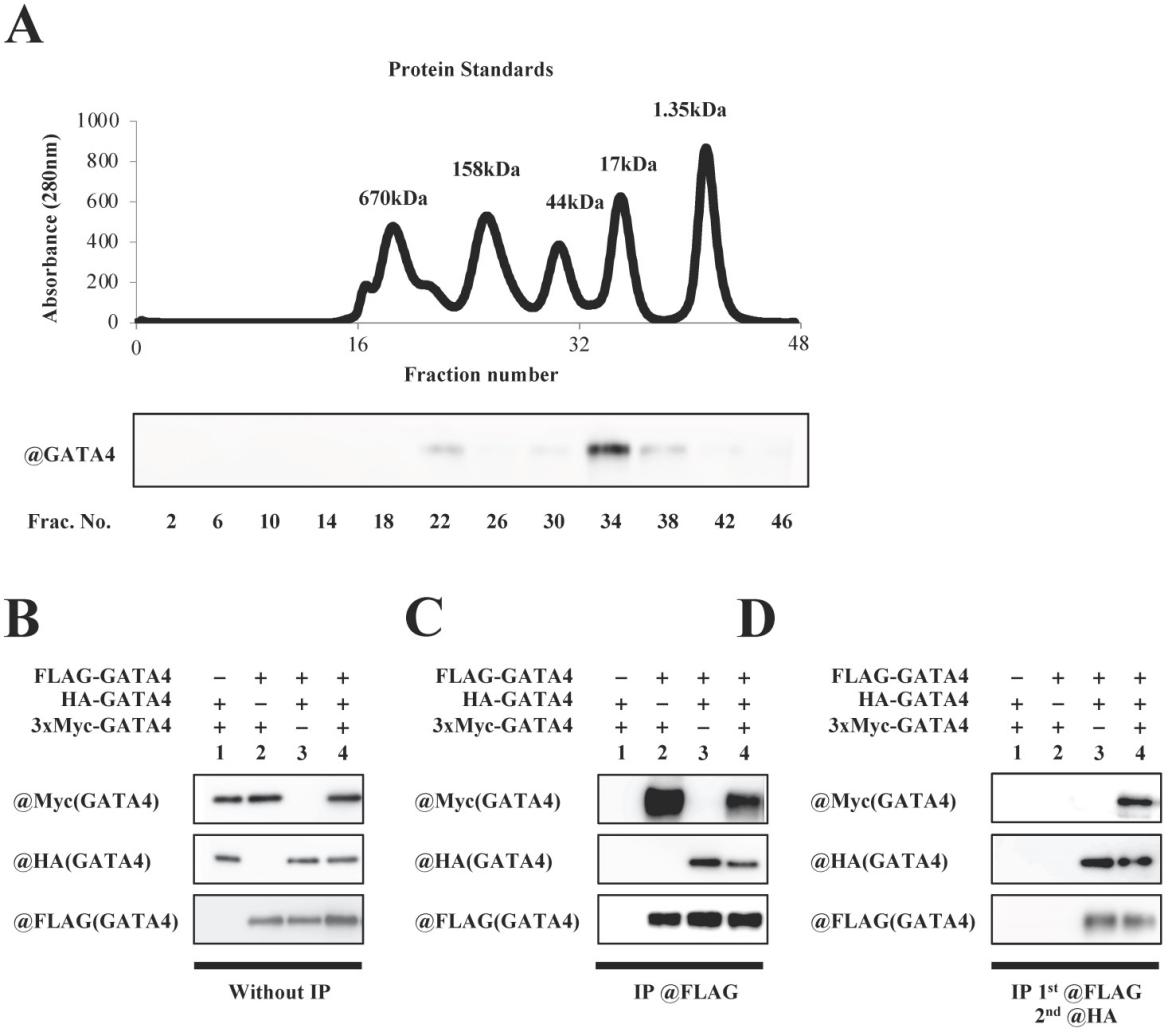
Multimeric formation of GATA4. (A) Full length GATA4 produced by *E. coli* was subjected to size exclusion chromatography. The upper panel shows a chromatogram of protein standards, and all fractions of the size exclusion chromatography were subjected to Western blotting with the anti-GATA4 antibody (lower panel). (B, C, D) HEK293T cells were co-transfected with FLAG-GATA4, HA-GATA4 and 3xMyc-GATA4. Nuclear extracts from these cells were subjected first to immunoprecipitation using ANTI-FLAG^®^ M2 Agarose Affinity Gel, and then the elution underwent a second immunoprecipitation with Anti-HA-tag pAb-Agarose. The immunoprecipitated samples were subjected to Western blotting with the anti-Myc, anti-HA, and anti-FLAG antibodies.

To investigate whether GATA4 forms a multimer, full length GATA4 produced by *E. coli* was subjected to size exclusion column chromatography, followed by Western blotting using an anti-GATA4 antibody. As shown in Fig. 4A, two GATA4 peaks were observed. The calculated molecular weights of the two peaks were 337 kDa and 29 kDa, respectively. Moreover, to determine whether GATA4 formed a multimer, HEK293T cells were co-transfected with FLAG-GATA4, HA-AGTA4 and 3xMyc-GATA4. Nuclear extracts prepared from these cells were immunoprecipitated with an anti-FLAG antibody, eluted with FLAG peptide, and then immunoprecipitated with an anti-HA antibody, followed by Western blotting using an anti-Myc, anti-HA, and anti-FLAG antibodies. (Fig.4B, C, D). As shown in Fig. 4D, Western blotting revealed a band of 3xMyc-GATA4, suggesting that GATA4 formed a multimer that had at least three subunits.

### Synthetic peptide containing residues 308-326 inhibited the multimerization and transcriptional activity of GATA4

**Figure 5:**
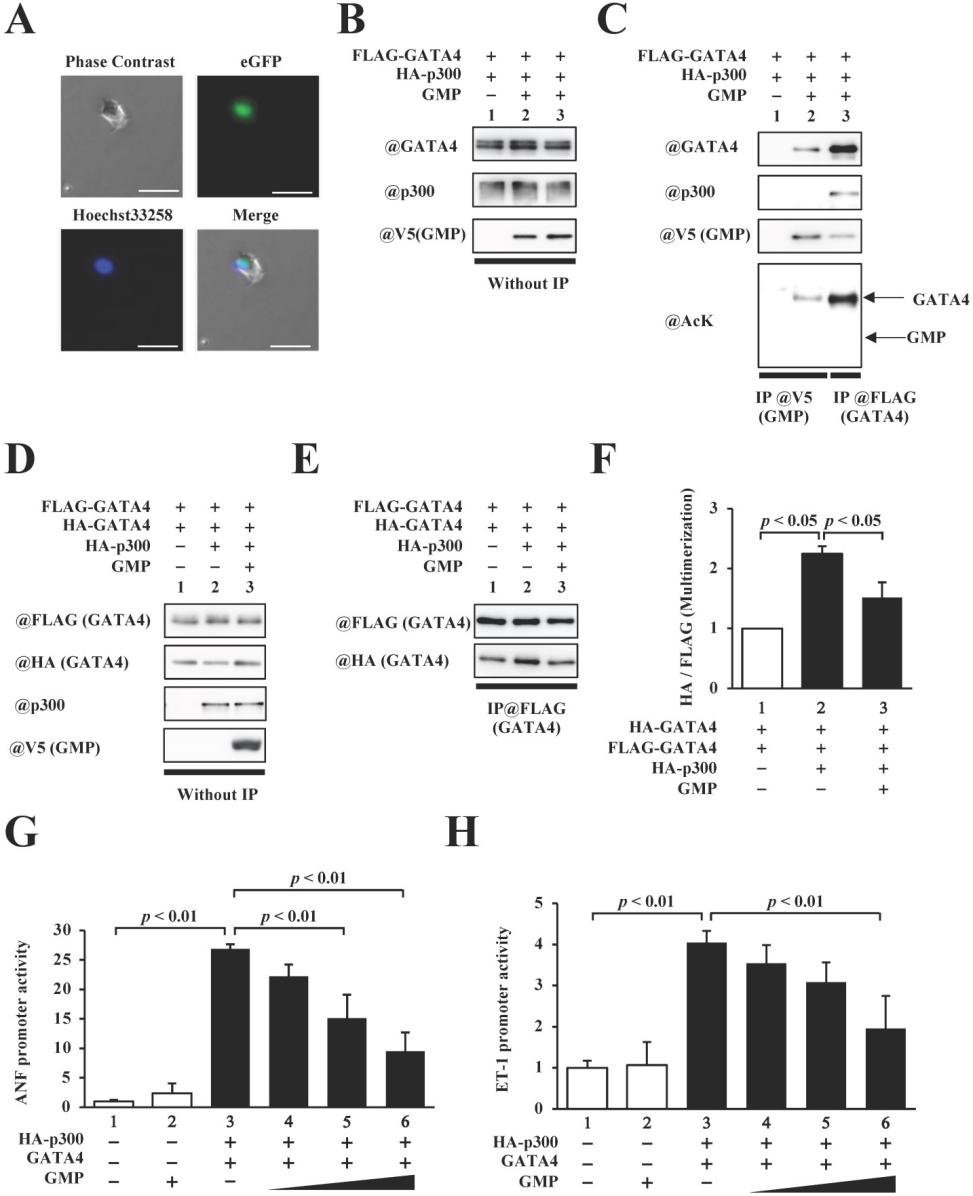
The homomultimerization and transcriptional activity of GATA4 were inhibited by GATA4 multimerization region peptide (GMP). (A) HEK293T cells were transfected with GMP and incubated for 48 h. Following incubation, the cells were stained with Hoechst 33258, and the green fluorescent protein (GFP) signal was detected. Scale bar, 50 µm. (B, C) The nuclear extracts from HEK293T cells expressing FLAG-GATA4, HA-p300, and GMP were immunoprecipitated with an anti-V5-tag antibody, and analyzed by Western blotting using anti-p300, anti-GATA4, anti-V5, or anti-acetyl lysine antibodies. (D, E) The nuclear extracts from HEK293T cells expressing FLAG-GATA4, HA-GATA4, HA-p300, and GMP were immunoprecipitated with an anti-FLAG antibody and analyzed by Western blotting using anti-HA and anti-FLAG antibodies. The signal levels of HA-GATA4 were measured relative to those of FLAG-GATA4. The HEK293T cells were co-transfected with pANF-luc (G) or pET-1-luc (H), and with pRL-SV40, FLAG-GATA4, HA-p300, and GMP. The relative promoter activity was calculated on the basis of the ratio of firefly to sea pansy luciferase activities. The results are presented as the mean ± S.E. of data obtained from three independent experiments.

In order to elucidate the role of GATA4 multimerization in the p300/GATA4 pathway, we synthesized an 82-residue peptide (GATA4 multimerization region peptide, GMP) containing residues 308-326 of GATA4, the nuclear localization sequences of the SV40 antigen, a 3xHA tag, and a V5 tag. HEK293T cells were transfected with this construct to determine the intracellular localization of GMP. Fluorescence imaging revealed that GMP was localized in the cellular nuclei (Fig. 5A).

Next, in order to investigate whether GMP bound to p300 and/or GATA4, the HEK293T cells expressing FLAG-GATA4 and p300 in the presence or absence of GMP (Fig. 5B) were subjected to analyses by immunoprecipitation assay and Western blotting. As shown in Fig. 5C, GMP retained its capacity to bind to GATA4 but not p300, which confirmed that GMP was not acetylated by p300.

In order to determine the effect of GMP on the multimerization of GATA4, we subsequently performed an immunoprecipitation assay and Western blotting. The results demonstrated that GMP significantly decreased the p300-mediated multimerization of GATA4 (Fig. 5D, E, F). Finally, in order to investigate whether GMP inhibited p300/GATA4-dependent transcriptional activation, reporter gene assays were performed with HEK293T cells, using ANF and ET-1 promoter-luciferase constructs. The results demonstrated that the co-expression of p300 and GATA4 synergistically enhanced the promoter activity of ANF and ET-1, and the co-expression of GMP significantly suppressed the enhanced promoter activities in a dose-dependent manner (Fig. 5G, H).

These results suggested that GMP interfered with the formation of a GATA4 multimer and prevented p300/GATA4-mediated transcriptional activation in HEK293T cells.

### GMP did not inhibit the p300-induced acetylation and DNA binding activities of GATA4

**Figure 6:**
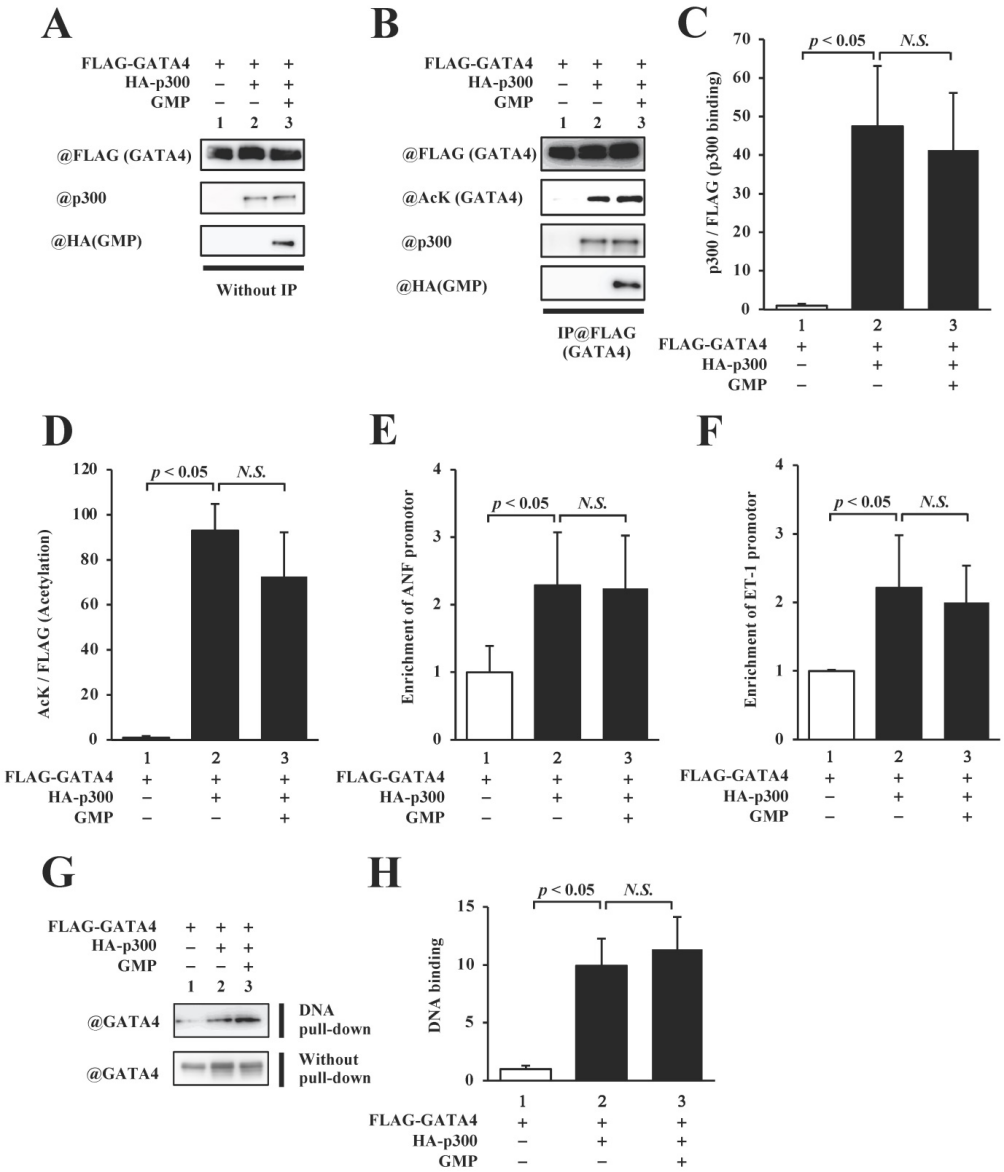
GMP did not inhibit the acetylation or DNA-binding activity of GATA4. The nuclear extracts from HEK293T cells expressing FLAG-GATA4, HA-p300, and GMP were immunoprecipitated with an anti-FLAG antibody, and analyzed by Western blotting using anti-FLAG, anti-acetyl lysine, anti-p300, or anti-HA antibodies (B). The signal levels for p300 and GATA4 acetyl lysine were quantified relative to those for FLAG (GATA4) (C, D). The HEK293T cells were co-transfected with pANF-luc (E), pET-1-luc (F), or with FLAG-GATA4, HA-p300, and GMP. The cells were then subjected to ChIP assay using an anti-FLAG antibody. The recruitment of GATA4 to the ANF or ET-1 promoter region on the DNA was analyzed by quantitative polymerase chain reaction (PCR). The results are presented as the mean ± S.E. of data obtained from three independent experiments. A biotin-labeled ET-1 probe was mixed with the nuclear extracts from HEK293T cells expressing FLAG-GATA4, HA-p300, and GMP, and then bound to streptavidin beads. The DNA-bound GATA4 was detected by Western blotting, and the signal levels for GATA4 were subsequently quantified (G, H).

In order to elucidate the mechanism by which the GMP-induced inhibition of GATA4 multimerization suppressed GATA4-dependent transcriptional activity, we measured the quantity of p300 that bound to GATA4 and quantified the levels of p300-mediated GATA4 acetylation. The overexpression of GMP did not reduce GATA4-p300 binding (Fig. 6B, C) or suppress the acetylation of GATA4 (Fig. 5B, D).

In order to determine whether the GMP-induced inhibition of GATA4 multimerization affected the recruitment of GATA4 by p300 to the promoter region of the target genes of GATA4, we performed a chromatin immunoprecipitation (ChIP) assay and an *in vitro* DNA pull-down assay. The results of the ChIP assay indicated that the overexpression of GMP did not suppress the recruitment of GATA4 to the ANF (Fig. 6E) or ET-1 (Fig. 6F) promoters, which contain a GATA element. The DNA pull-down assay further suggested that p300 increased the recruitment of GATA4, and GMP did not suppress p300-induced GATA4 recruitment (Fig. 6G and H). These results indicated that GMP inhibited the multimerization of GATA4, but did not inhibit the acetylation or DNA binding activity of GATA4. These results demonstrated that GMP inhibited the transcriptional activity of GATA4 by suppressing the homomultimerization of GATA4.

### Inhibition of GATA4 multimerization suppressed phenylephrine (PE)-induced hypertrophic response in cultured cardiomyocytes

**Figure 7:**
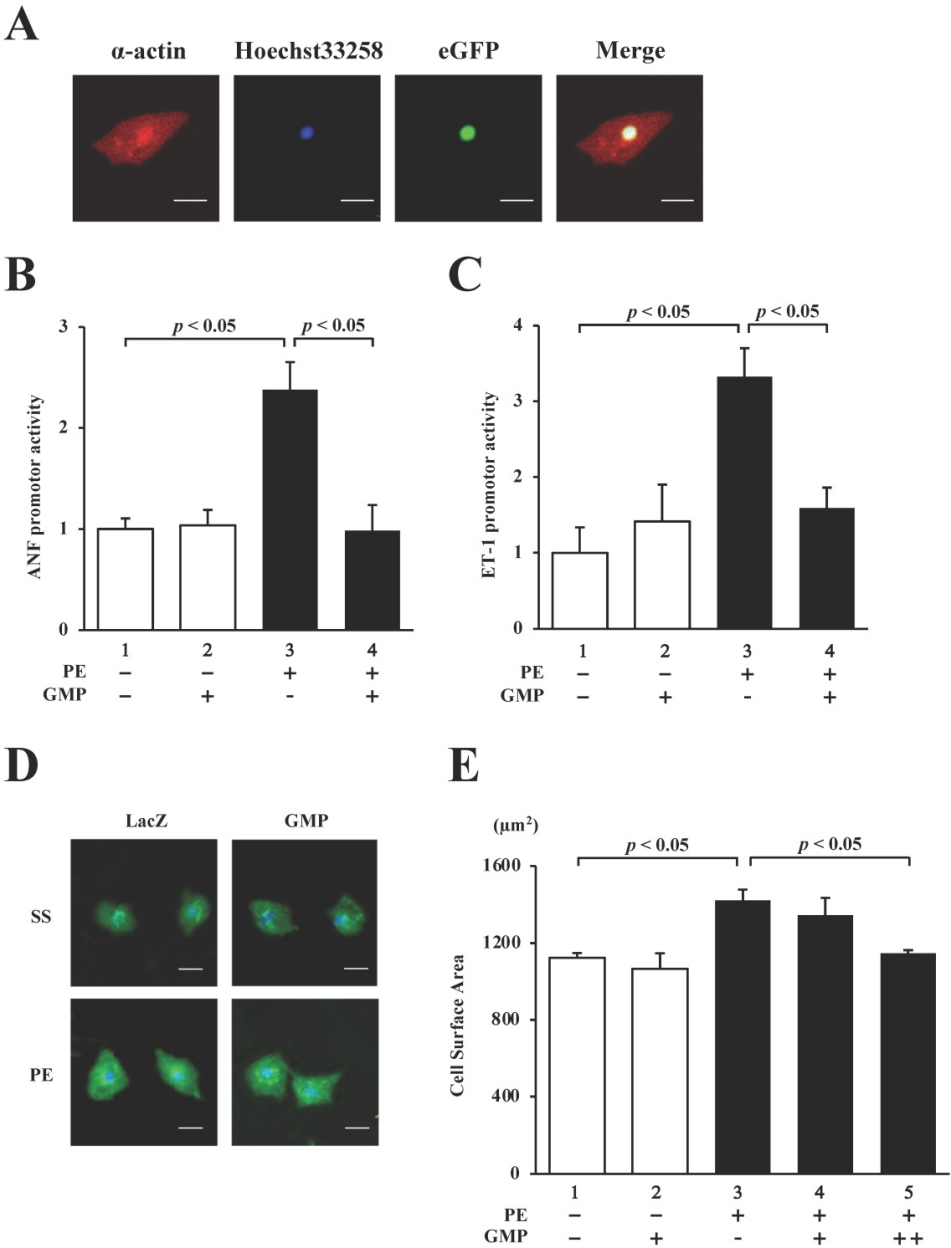
GMP inhibited the PE-induced transcriptional activity of GATA4 and cardiomyocyte hypertrophy. (A) Immunofluorescence staining was performed using an anti-α-actin antibody. The cardiomyocytes were co-transfected with pANF-luc (B) or pET-1-luc (C), together with GMP. The cells were incubated with 30 μM PE or left untreated for 48 h. The relative promoter activity was calculated from the ratio of firefly to sea pansy luciferase activity. The results are presented as the mean ± S.E. of data obtained from three independent experiments. The cardiomyocytes were immunostained with the anti-α-actin antibody (red signals). Representative images obtained from immunostaining studies (D). The surface area of the cells was measured using a Cellomics^TM^ ArrayScan® V^TI^ System (E). The scale bars represent 20 μm. The data are expressed as the mean ± S.E. (n = 3).

In order to confirm whether GMP localizes in the nucleus of cultured neonatal rat cardiomyocytes (NRCMs), the cardiomyocytes were transduced with GMP using a lentiviral vector and subjected to immunocytochemical staining with anti-α-actin antibody (red signal in Fig. 7A). The results demonstrated that GMP (green signal in Fig. 7A) was localized in the nuclei.

We subsequently performed reporter assays with the NRCMs to investigate whether GMP inhibits the PE-induced activation of the hypertrophic response. The results revealed that the overexpression of GMP significantly inhibited the PE-induced activation of the ANF (Fig. 7B) and ET-1 (Fig. 7C) promoters.

Finally, in order to investigate whether GMP inhibits PE-induced cardiomyocyte hypertrophy, GMP and LacZ (negative control) lentiviruses were introduced into NRCMs. The cells were stimulated with 30 µM PE or left unstimulated for 48 h, and subsequently stained with an anti-α-actin antibody. As shown in Fig. 7D and E, the sizes of the NRCMs stimulated with PE were higher than those of the untreated cells. The results also demonstrated that the changes induced by PE were inhibited by the transduction of GMP. These results confirmed that GMP inhibited hypertrophic responses in NRCMs, suggesting that the homomultimerization of GATA4 plays a key role in hypertrophic responses.

## Discussion

GATA4 is a zinc finger transcription factor that plays an important role in the development and progression of heart failure 8, 18, 23. It has been reported that the transcriptional mechanism of GATA4 is regulated by its binding to co-factors and post-translational modifications 9, 24, 25. However, the mechanism by which these proteins regulate the transcriptional activity of GATA4 remains unelucidated. This study revealed that GATA4 formed multimers that had three or more subunits. The fractions of GATA4 detected by size exclusion chromatography were at 29 kDa and 337 kDa. The calculated molecular weight of the second peak near the 34th fraction was less than 45 kDa, and the absence of a GATA4 peak in the posterior fraction suggested that this second peak was a monomer. Furthermore, the calculated molecular weight of 337kDa for a peak around the 22nd fraction indicated the presence of a multimer approximately an octamer in size. p53 is a well-known tetrameric protein. A previous study using size exclusion column chromatography reported that the peak of p53 was around 440 kDa 26. Size exclusion column chromatography separates proteins based on their size but not their molecular weight. Therefore, the molecular weight calculated from size exclusion column chromatography in that study may not have been accurate. That study used sucrose gradient centrifugation to accurately determine that p53 was a tetramer. This indicates that size exclusion chromatography is not accurate enough to determine whether a GATA4 multimer is an octamer from calculated molecular weight; therefore, other methods such as sucrose density gradient centrifugation should be performed to determine the total number of subunits in a GATA4 multimer. Previous research has also shown that the proteins histone and retinoschisin form octamers 27. It has also been reported that transcription factors such as MADS-domain proteins form multimers. The role of multimerization in these proteins is still unknown, but it is thought to be important for DNA binding and protein-protein interaction 28. GATA4 has various modes of action associated with DNA-protein interactions 7, suggesting that multimerization may play an important role in these modes of actions.

The multimerization of transcription factors plays an important role in the regulation of transcriptional activity. For instance, p53 forms homotetramers such as STAT3, and CREB forms homodimers and heterodimers that bind to DNA and regulate transcriptional activity. The post-translational modification of these transcription factors, including phosphorylation and acetylation, play important roles in their multimerization 29-32. In the present study, we examined multimerization and the transcriptional activation of GATA4 by p300-induced acetylation. P300-induced acetylation of GATA4 increased its homomultimerization (Fig. 5D-F), DNA binding activity (Fig.6G), and transcriptional activity (Fig. 5G, H). In addition, GATA4 multimerization and the promoter activity of hypertrophic genes were increased in a p300 dose-dependent manner (Fig. 3A-E). These results suggest that the amount of GATA4 multimer formation is related to the amount of pro-hypertrophic transcriptional activity. Furthermore, inhibition of GATA4 homomultimerization suppressed transcriptional activation by p300 (Fig. 5D-H). These results indicate that GATA4 forms a multimer due to post-translational modifications in a manner similar to p53, STAT3, and CREB, and that the multimerization of GATA4 regulates its transcriptional activity. This suggests that GATA4 multimerization is a key mechanism of its transcriptional activation.

Previous studies have reported that the transcription of hypertrophic response genes increases in transgenic mice with a cardiac-specific overexpression of GATA4, leading to cardiac hypertrophy and heart failure 33. It has been previously reported that homo-GATA4 and hetero-GATA4 knockout murine embryos suffer early developmental arrest owing to functional deficiencies in the extraembryonic visceral endoderm 34, 35. These studies indicate that GATA4 regulates cardiogenesis and cardiac hypertrophy 36. GATA4 regulates its DNA binding properties, transcriptional activity, and binding to transcriptional cofactors, not only via alterations in the expression level, but also through post-translational modifications, including acetylation and phosphorylation 18, 37. This has been observed in the inhibition of GATA4 acetylation by curcumin, a p300 acetyltransferase inhibitor that suppresses the transcriptional activity of GATA4 and prevents hypertrophic responses in cardiomyocytes and the development of heart failure 9, 10, 11. It has been further reported that the DNA binding activity of GATA4 does not increase in GATA4 knock-in mice in which the serine 105 phosphorylated by ERK is mutated to alanine. These mice also exhibit suppressed cardiac hypertrophic responses and development of heart failure 38. The present study elucidated that the homomultimerization of GATA4 regulates its transcriptional activity, and that the inhibition of multimerization suppresses the increased transcriptional activity of GATA4 and cardiomyocyte hypertrophy. This indicated that the multimerization of GATA4 plays a crucial role in the hypertrophic response in cardiomyocytes. Therefore, the inhibition of GATA4 homomultimerization can serve as a potential therapeutic strategy for heart failure, as can the inhibition of GATA4 acetylation and phosphorylation.

The signaling pathways of cardiomyocyte hypertrophy include ERK/JNK/p 38 (MAPK), calcineurin-NFAT, JAK/STAT, and p300/GATA4 8, 39-41. Stimulation by PE activates these pathways. In the present study, a small peptide containing the GATA4 multimerization region,GMP, completely suppressed PE-induced cardiomyocyte hypertrophic responses. These results suggest that GMP may not only inhibit GATA4 multimerization but also block other protein interactions in these signaling pathways. Many of the proteins that bind to GATA4 and work together such as MEF, Nkx2.5, NFATc4, RACK1, and Cdk9 bind near the C-terminal zinc finger domain, including the multimerization region 42-46. It is possible that GMP suppresses GATA4 transcriptional activity by inhibiting GATA4 from binding to its binding proteins, thereby inhibiting cardiomyocyte hypertrophy. In this study, the interaction between GATA4 and p300 was not inhibited by GMP, but further proteomic analysis of GMP binding proteins is needed to investigate the effects of GMP.

The members of the GATA family can be categorized into two subgroups based on their developmental and pathological functions, namely, GATA1/2/3, which partake in the hematopoietic system, and GATA4/5/6, which partake in the functions of endodermal tissues 6, 47. The zinc finger domain necessary for DNA binding is highly conserved in the GATA family, but only approximately 30% of the other regions are conserved in this family. Additionally, gene promoters and enhancers, including Lmo2 and Uros, which are transcriptionally regulated by GATA1/2/3, contain two GATA sequences, including tandem-GATA sequences (GATA ・・・ GATA), reverse palindromic GATA sequences (GATA ・・・ TATC), and palindromic GATA sequences (CTAT ・・ GATA). These sequences are vital to the transcriptional regulation of GATA1/2/3 48-50. The GATA1 homodimer binds the tandem-GATA sequence, while the GATA3 homodimer binds the reverse palindromic GATA sequence 49, 51. It has been reported that the NRPL motif (Asn-Arg-Pro-Leu) is an important dimerization region in GATA1 and GATA3 (GATA1: residues 292-295; GATA3: residues 351-354) 21, 51. The multimerization region of GATA4 (residues 308-326) identified in the present study did not contain the NRPL motif. Of the 19 amino acids in the multimerization region of GATA4 (residues 308-326), only 10 are conserved in GATA1, 11 in GATA3, and as few as 9 are conserved in the other members of the GATA family. The gene promoters and enhancers, including ANF and ET-1, which are transcriptionally regulated by GATA4, contain a single GATA sequence (・・・ GATA・・・), unlike the target genes of GATA 1/2/3, which contain two GATA sequences 52, 53. This suggests that, unlike GATA1 and GATA3, only one GATA4 heterodimeric complex binds to DNA. Furthermore, there are no reports of the multimerization of GATA1 and GATA3. Taken together, this indicates that the multimerization patterns of GATA1/2/3 and GATA4 are different.

The E1A binding protein p300 partakes in transcriptional activation via its acetyltransferase activity and also by serving as a scaffold for the assembly of multiprotein complexes or by acting as a bridge allowing the binding of proteins to the transcriptional machinery 22. In order to elucidate which of these functions of p300 are essential for GATA4 homomultimerization, we used the GATA4 m456A point mutant, which is not acetylated by p300, and the p300 AS point mutant, which is deficient in acetyltransferase activity 9, 10. The results demonstrated that the p300-induced acetylation and multimerization of GATA4 did not increase in the experiments with GATA4 m456A or p300 AS. This indicated that the acetylation activity of p300, and not its scaffold and bridge functions, plays an important role in GATA4 homomultimerization. We are presently investigating whether compounds that inhibit the acetyltransferase activity of p300, such as curcumin, also inhibit the acetylation and multimerization of GATA4.

Although the structure of the GATA4-DNA complex is unknown at present, the structures of GATA1 and GATA3 have been reported. The side chains of arginine, especially those of R305 and R307 of GATA1 and R364 and R366 of human GATA3 (equivalent to the side chains of R317 and R319 of GATA4), which are located in the multimerization region, insert into the DNA double helix and interact with the DNA 51, 54. The amino acids near the acetylation site of GATA4 extend the side chain to the solvent side opposite to the DNA, but do not interact with the DNA. This suggests that the acetylation sites of GATA4 (K311/K318/K320/K322) do not directly interact with the DNA, unlike those of GATA1 and GATA3. The multimerization region of GATA4 contains an acetylation site, which includes numerous arginine and lysine residues, and has a positive surface charge. Acetylation involves the transfer of acetyl groups to lysine residues, leading to the neutralization of the positive charges of the residues. This suggests that the acetylation of GATA4 by p300 weakens protein-protein repulsion and promotes the homomultimerization of GATA4 55.

It has been reported that phosphorylation and acetylation increase the stability of p53, and that tetramerization is important for p53-DNA binding 56. STAT3 is phosphorylated to form a dimer that translocates to the nucleus and activates the transcription of target genes 57. It is known that post-translational modification of transcription factors is involved in multimerization and DNA binding. In this study, we first examined the relationship between the acetylation and multimerization of GATA4. The acetylation of GATA4 by p300 increased GATA4 multimerization (Fig. 3). GATA1, another member of the GATA family, is acetylated by p300 in the same manner as GATA4. Specifically, the GATA1 lysines K312 and K315 are acetylated by p300 58. These acetylated lysine residues enter the pores in the bromodomain of BRD4 59. These GATA1 lysines, which are also acetylated by p300 and are involved in protein-protein interactions, correspond to the multimerization region of GATA4, suggesting that GATA4 acetylation is important for protein-protein interaction. In the results of size exclusion chromatography using recombinant GATA4, a low level of unacetylated GATA4 multimer was also detected in equilibrium (Fig.4A, B). However, the first step in the increase in the amount of GATA4 multimerization was the acetylation of monomeric GATA4 by p300. Subsequently, stabilization of the GATA4 multimer may have been improved, resulting in an increase in the amount of multimeric GATA4.

This study has also eliminated the relationship between the formation of GATA4 multimerization and DNA binding. We investigated the inhibition of GATA4 multimerization by GMP and the acetylation and DNA binding of GATA4. While GMP did inhibit GATA4 multimerization (Fig. 5D-F), it had no effect on the acetylation or DNA binding of GATA4 (Fig. 6). These results indicated that GATA4 multimerization was independent of DNA binding and suggested that the acetylation of GATA4 was important for DNA binding. A previous report on the crystal structure analysis of GATA3 suggested that the region that is acetylated by p300 acts as an anchor to strengthen DNA binding (54). As shown in Supplement Fig. 1, we found that the DNA binding of GATA4 required not only the zinc finger domain but also an acetylation site, suggesting that the acetylation of lysine residues by p300 strengthens the binding between DNA and the acetylation site of GATA4. Acetylated monomeric and multimeric GATA4 were also bound to DNA (Fig. 5D-F, Fig. 6). These results suggested that multimerization and DNA binding are unrelated. However, it is not clear whether multimerization or DNA binding occur first. The above findings suggested that GATA4 requires post-translational modifications for multimerization (like p53 and STAT3), but not multimerization for DNA binding (like p53) or for nuclear translocation (like STAT3). Further study is required to determine whether the multimerization or the DNA binding of GATA4 occur first, and to elucidate in detail the role of multimerization.

Several transcription factors undergo post-translational modifications that induce their multimerization and DNA binding function, thereby increasing their transcriptional activity. For instance, estrogen receptor α, which has a zinc finger domain, is phosphorylated by protein kinase A and activates transcriptional activity. This phosphorylation enhances the dimerization of estrogen receptor α and its DNA binding activity 58-60. In this study, we used GMP, to elucidate whether the multimerization of GATA4 plays a role in its DNA binding activity, transcriptional activity, and hypertrophic responses in cardiomyocytes. The results demonstrated that GMP was localized in the nucleus and bound to GATA4 but not p300. GMP also suppressed p300-induced GATA4 multimerization and the transcriptional activity of ANF and ET-1, without affecting the acetylation of GATA4 or its DNA binding activity. Additionally, the suppression of GATA4 multimerization repressed transcriptional activity and cardiomyocyte hypertrophy without altering the binding of GATA4 to the promoters of the target genes of GATA4. This suggested that the homomultimerization of GATA4 plays an important role in its transcriptional activation and hypertrophic responses in cardiomyocytes.

GATA6, a homolog of GATA4, has been detected in many tissues. In the heart, GATA6, like GATA4, is involved in the differentiation and induction of cardiomyocytes and in their hypertrophic response 37, 61. GATA6 also interacts with GATA4 and is involved in the transcriptional activation of the ANF and BNP promoters 62, although some hypertrophic genes, such as β-MHC, have a lower affinity for DNA than GATA4 does 63. The homology of GATA6 with GATA4 is very high, reaching 89.5% (17/19) in the multimerization region (GATA4 308-326, GATA6 476-494) 64, 65, and distinct amino acids have a similar in structure in the two: R310 and L324 of GATA4 are analogous to the basic and branched amino acids K478 and I492 of GATA6. In addition, GATA6 has been reported to bind to p300 and increase transcriptional activity 62. Therefore, we hypothesize that GATA4-GATA6 heteromultimer formation activates transcription via the same mechanism as GATA4 homomultimer formation. We also hypothesize that GMP binds to GATA4 and GATA6, inhibits GATA4-GATA6 binding, suppresses an increases in ANF and ET-1 promoter activity, and suppresses the hypertrophic response. Taken together, the above suggests that GMP not only inhibits the binding of GATA4 to GATA4 but also may inhibit the binding of GATA4 to GATA6, indicating that the inhibition of hypertrophic response is additive. We will further investigate whether GMP inhibits the formation of GATA4-GATA6 heterodimers and how that may affect hypertrophic response in cardiomyocytes.

The GATA4 binding proteins, including p300, RACK1, CDK9, TBX5, Nkx2.5, MEF2, SRF, NFATc4, and HAND2, bind with the C-terminal zinc finger domain of GATA4 (residues 256-326), which includes the multimerization region 13, 14, 17, 37, 44, 45, 66, suggesting that numerous cofactors are concentrated near this region. The results of this study revealed that GMP completely inhibited the PE-induced hypertrophic response in the cardiomyocytes. Stimulation by PE activated various cardiac hypertrophy pathways, and GMP completely inhibited the cardiomyocyte hypertrophic response, suggesting the possibility that GMP inhibited protein-protein interactions near the multimerization region. That is, GMP not only suppressed the homomultimerization of GATA4 but also inhibited the formation of GATA4-cofactor heterodimers. In addition, the GATA family catalyzes chromatin looping, in which a loop of nuclear chromatin is formed, and this has been reported to play a role in transcriptional regulation. GATA transcription factors also exhibit synthetic activation, in which a transcriptional complex is formed, following which, transcription is activated 7. In order to elucidate the mechanism linking GATA4 multimerization with transcriptional activation and cardiomyocyte hypertrophy, we intend to investigate the effect of alterations in DNA looping and the role of GATA4 binding proteins in heart failure by chromosome conformation capture assays and proteomic analyses in the future.

It has been reported that both the expression level and the HAT activity of p300 are increased in hearts with ischemic, diluted, or unspecified end-stage cardiomyopathy compared with hearts without heart failure (67). Therefore, GATA4 multimerization may be increased in the hearts of human heart failure patients, together with increased GATA4 acetylation. Protein homomultimers have been detected by immunoprecipitation followed by Western blotting using tagged proteins, by native polyacrylamide gel electrophoresis, and by electrophoretic mobility shift assay (68-70). It has been reported that GATA4 interacts with many proteins, including MEF, NKx2.5, NFATc4, RACK1, and Cdk9 (14, 17, 46, 53, 71). Therefore, it is difficult to determine whether GATA4 is multimerized based on molecular weight after separating the complex in a non-native state. In this study, the multimerization of GATA4 was detected by immunoprecipitation using GATA4 tagged with HA and FLAG tags. As cardiomyocytes are non-dividing, it is difficult to establish cardiomyocyte cell lines following gene transfer, which makes it difficult to tag endogenous GATA4. Moreover, the overexpression of GATA4 in cardiomyocytes itself induces cardiomyocyte hypertrophy 33. In order to resolve these issues, we will perform immunoprecipitation and Western blotting assays with knock-in mice generated to express the two required kinds of GATA4 with protein tags such as FLAG and Myc. In addition, to investigate whether the multimerization of GATA4 increases or decreases in heart failure, we intend to perform immunoprecipitation and Western blotting assays using the cardiac tissues of mice that have undergone heart failure-inducing surgery.

It has been reported that curcumin, which inhibits the acetyltransferase activity of p300, suppresses the acetylation not only of GATA4 but also of histone, and thereby suppresses heart failure 11, 12, 72. We hypothesize that curcumin inhibits GATA4 acetylation, thereby inhibiting GATA4 multimerization. On the other hand, GATA4 is regulated by phosphorylation via ERK and Akt. Curcumin alone cannot suppress these phosphorylation signals and may not be sufficient to suppress GATA4 activation. Therefore, it may be possible to obtain additive effects by combining curcumin with GMP. Further research is needed to determine whether a co-effect can be obtained with this combination. At present, there are no compounds that are known to inhibit GATA4 multimerization. We plan to search for inhibitors of GATA4 multimerization by developing assays using the yeast two-hybrid method and screening compound libraries.

In conclusion, this study demonstrates that the inhibition of GATA4 multimerization suppresses hypertrophic responses in cardiomyocytes. The results of this study suggested that the inhibition of GATA4 homomultimerization can serve as a possible therapeutic strategy and preventive measure for heart failure. The study further revealed that it may be possible to develop GATA4-specific drugs and avoid the potential side effects triggered by the inhibition of GATA1/2/3 dimerization. This can be achieved by targeting the multimerization region of GATA4, which is not highly conserved in the rest of the GATA family, instead of inhibiting the zinc finger domain, which is highly conserved among the GATA family of transcription factors. The development of drugs that specifically inhibit GATA4 multimerization is a promising novel approach to the treatment of heart failure.

## Material and Methods

### Plasmid constructs and mutants

The expression vectors for full length pDEST15 GATA4 and residues 1-179, 180-326, 256-326, and 325-440 of pDEST15 GATA4, pGEX6P-1 GATA4, pCMV-p300, p300 AS, pcDNA3.2 FLAG-GATA4, FLAG-GATA4 m456A, pcDNA3.2 HA-GATA4, pANF-luc, and pET-1-luc have been previously described 8, 45, 52, 72. The gateway destination vectors for residues 180-255, 256-295, 296-326, 296-307, and 308-326 of pDEST15 GATA4 were constructed using inverse PCR using the full length pDEST15 GATA4 as the template for deletion mutations. The 3xMyc tag sequences, which were purchased from Integrated DNA Technologies (Coralville, IA, United States), and the amplified GATA4 sequence were cloned into an entry vector and then subcloned into pcDNA3.2/V5-DEST using Gateway Technology (Thermo Fisher Scientific, Waltham, Massachusetts, United States). The 308-326 fragment of GATA4 tagged with HA and V5 sequences was purchased from FASMAC (Kanagawa, Japan) and amplified by PCR. The PCR product of GMP was cloned into an entry vector and subcloned into pcDNA3.2/V5-DEST and pLenti6.3/V5-DEST using Gateway technology (Thermo Fisher Scientific). The FLAG-GATA4 K311A/K318A/320A/322A (m456A) mutants used in this study can interact with p300 but are not acetylated by p300 8. The HA-p300 W1466A/Y1467S (AS) mutants used herein can interact with GATA4 but do not show acetyltransferase activity 9, 10.

### *In vitro* binding assay

An ^35^S-labeled protein was prepared using a TnT^®^ Quick Coupled Transcription/Translation System (Promega, Madison, WI, USA), following the manufacturer's instructions. The GST fusion proteins were expressed in *Escherichia coli* BL21 DE3 and immobilized on Glutathione-Sepharose 4B beads (Cytiva, Tokyo, Japan). The beads were mixed with ^35^S-labeled protein and incubated for 2 h at 4°C. The beads were subsequently washed five times with wash buffer (20 mM Tris pH 8.0, 2.5 mM MgCl_2,_ 100 mM KCl, 5% glycerol, and 0.1% Tween20), resuspended in sample buffer, analyzed by SDS-PAGE, and transferred to a Poly vinylidene fluoride (PVDF) membrane (Millipore, Billerica, Massachusetts, United States). The GST fusion proteins were detected by Coomaisse brilliant blue staining. The PVDF membrane was subsequently dried and exposed to a BAS-IIIS imaging plate (Fujifilm, Tokyo, Japan) for 3 h. The ^35^S-labeled proteins were detected using a BAS2000 bio-imaging analyzer (Fujifilm).

### Size exclusion chromatography

GST-GATA4 was expressed in *Escherichia coli* BL21 DE3 and immobilized on Glutathione-Sepharose 4B (Cytiva). The beads were mixed with HRV3C protease. The GST-tag was removed from GST-GATA4, and the GATA4 protein was obtained. The purified GATA4 was passed through a size-exclusion column, Superdex200 Increase 10/300 GL (Cytiva), equilibrated with eluent buffer (20 mm HEPES-NaOH, pH 7.4, and 100 mM NaCl).

### Cell culture, transfection, and dual-luciferase assay

HEK293T cells were maintained in Dulbecco's Modified Eagle's Medium (Nacalai Tesque, Kyoto, Japan) as previously described 45. The HEK293T cells were transfected with plasmid DNA by using polyethyleneimine (PEI). Primary cultured neonatal rat cardiomyocytes were prepared as previously described (73). All animal experiments complied with the guidelines on animal experiments of the University of Shizuoka and were performed in accordance with protocols approved by University of Shizuoka Ethics Committee (number 176278). The hearts of neonatal 2-day-old SD rats were removed and minced, then underwent enzymatic dissociation with 2 mg/ml pancreatin (Sigma-Aldrich) and 2 mg/ml collagenase type 2 (Worthington Industries, Columbus, OH, USA). Cells were separated into cardiomyocytes and cardiac fibroblasts using the Percoll gradient method 74. After separation, cardiomyocytes were cultured at a density of 3.0 × 10^5^ cells/well on a 6-well plate for dual-luciferase assay or 6.0 × 10^3^ cells/well on a 48-well plate for immunofluorescence staining in Dulbecco's Modified Eagle's Medium (DMEM) consisting of 10% (vol/vol) fetal bovine serum (FBS, Corning, Corning, NY, USA or Thermo Fisher Scientific) at 37°C with 5% CO_2_. Twenty-four hours after plating, cells were washed twice with DMEM without FBS before being replaced with fresh media. The primary neonatal rat cardiomyocytes were transfected with the indicated amounts of DNA by using Lipofectamine LTX with Plus Reagent (Thermo Fisher Scientific), as previously described 75. A luciferase reporter assay was performed using HEK293T cells and primary neonatal rat cardiomyocytes, as previously described 8, 48. The luciferase activity was measured using a dual luciferase reporter assay system (Promega), according to the manufacturer's instructions.

### Immunoprecipitation and Western blotting assay

Nuclear extracts were prepared from HEK293T cells, and immunoprecipitation studies and Western blotting were performed as previously described 75. For the immunoprecipitation studies, an ANTI-FLAG® M2 Agarose Affinity Gel (Sigma-Aldrich, St. Louis, Missouri, United States), Anti-HA-tag pAb-Agarose (Medical & Biological Laboratories, Tokyo, Japan), anti-HA-tag mAb (Medical & Biological Laboratories), and normal murine IgG (Jackson ImmunoResearch Laboratories, West Grove, Pennsylvania, United States) were used. Elution of the proteins was carried out with 100 μg / ml FLAG peptide (Sigma-Aldrich) or 0.1 M Glycine pH 2.5.

For Western blotting, rabbit polyclonal acetylated lysine antibody, (Cell Signaling Technology, Danvers, Massachusetts, United States), goat anti-GATA4 polyclonal antibody, murine anti-GATA4 monoclonal antibody, rabbit anti-p300 polyclonal antibody, murine anti-p300 monoclonal antibody, rabbit anti-V5-probe polyclonal antibody (Santa Cruz Biotechnology, Dallas, TX, USA), murine monoclonal FLAG (M5) antibody (Sigma-Aldrich), murine anti-FLAG monoclonal antibody, murine anti-HA monoclonal antibody, and murine anti-Myc monoclonal antibody (Medical & Biological Laboratories) were used. The levels of the bands were detected with an Amersham Imager 680 (Cytiva) and quantified using ImageJ software, version 1.52q.

### ChIP assay

In this study, the ChIP assays were performed as previously described 45, 76. Briefly, the genomic DNA and nuclear proteins were fixed with formalin, and the extracts were sonicated and subsequently immunoprecipitated with ANTI-FLAG® M2 agarose affinity gel or control murine IgG. The immunocomplexes were then captured with protein G beads (GenScript Biotech Corporation, Piscataway, New Jersey, United States). The precipitates were washed four times in a low stringency buffer, following which the DNA was purified by phenol-chloroform extraction and precipitated with ethanol. In order to detect the ANF or ET-1 promoters that contained a GATA site, the collected DNA was subjected to PCR analysis with a thermal cycler, using specific primers for ANF or ET-1 promoters. The sequences of the primers were as follows: 5′- GACTGATAACTTTAAAAGGG-3′ (forward primer for the ANF promoter), 5′-TCTCTCTCAGCCTTTGTCCG-3′ (reverse primer for the ANF promoter), 5′-ATTGTCAGACGGCGGGCGTC-3′ (forward primer for the ET-1 promoter), and 5′-GTCTGACTTGGACAGCTCTC-3′ (reverse primer for the ET-1 promoter). Quantitative real-time PCR was performed using KOD SYBR qPCR Mix (Toyobo, Osaka, Japan), and the products were analyzed using a LightCycler 96 Real-Time PCR System thermal cycler (Hoffmann-La Roche, Basel, Switzerland).

### DNA binding assay

Nuclear extracts were prepared from HEK293T cells and mixed with a biotin-labelled ET-1 probe (sense, 5′-BioCCTCTAGAGCCGGGTCTTATCTCCGGCTGCACGTTGC-3′; anti-sense, 5′-GCAACGTGCAGCCGGAGATAAGACCCGGCTCTAGAGG-3′) in DNA binding buffer (0.3 mg/ml bovine serum albumin (BSA), 0.1 mg/ml salmon sperm, 20 mM Hepes pH 7.9, 1.5 mM MgCl_2_, 400 mM NaCl, 0.2 mM EDTA, 25% glycerol, 0.02% Tween10) and incubated for 2 h at 4°C. Streptavidin Sepharose High Performance Beads (Cytiva) were added to the protein-DNA mixtures and incubated for 2 h at 4°C. The beads were then washed four times with wash buffer, resuspended in 0.1 M glycine (pH 2.5 at 4°C for 5 min), and subsequently analyzed by Western blotting.

### Production of lentiviral vector-expressing GMP

HEK293T cells were co-transfected with pLp1 (gag/poly), pLp2 (rev), pLp/vesicular stomatitis virus G, and plenti6.3-GMP, and subsequently packaged into a pseudotyped lentivirus as previously described 45. The viral supernatants were harvested after 48 h of transfection and filtered through a 0.45-μm filter. The supernatants were then added to the cardiomyocytes and incubated for 48 h.

### Immunofluorescence staining and measurement of surface area of cardiomyocytes

The immunofluorescence staining of cultured cardiomyocytes was performed as previously described 11, 46. Briefly, the cardiomyocytes were cultured in a 24-well multiwall plate (Corning, Corning, New York, United States) and stained with monoclonal anti-α-actinin (sarcomeric) antibody (Sigma-Aldrich), and Alexa Fluor 555-conjugated anti-mouse IgG (Invitrogen, Carlsbad, CA, USA). Hoechst 33258 (Dojindo Laboratories, Kumamoto, Japan) was used for nuclear staining. The surface area of the cardiomyocytes was automatically calculated using an ArrayScan system (Thermo Fisher Scientific) based on a positive indication for Alexa Fluor 555 (Thermo Fisher Scientific).

### Statistical analyses

The values are presented as the mean ± standard error (S.E.) of data from at least three independent experiments. The statistical comparisons were performed using the Analysis of Variance test with the Tukey-Kramer test. Statistical significance was considered at *p* < 0.05.

## Supplementary Material

Supplementary figure and methods.
