# Supplementary material for: Multimerization of the GATA4 transcription factor regulates transcriptional activity and cardiomyocyte hypertrophic response
Source: Int J Biol Sci. 2022 Jan 1;18(3):1079–95. doi: 10.7150/ijbs.65664 (PMC8771830; doi:10.7150/ijbs.65664)
Supplement: Supplementary file 1 — Supplementary figure and methods. [file ijbsv18p1079s1.pdf]

SUPPLEMENTARY MATERIAL

Multimerization of the GATA4 transcription factor regulates transcriptional activity and cardiomyocyte hypertrophic response

Satoshi Shimizu[1,2], Yoichi Sunagawa[1,2,3], Yasufumi Katanasaka[1,2,3], Masafumi Funamoto[1,2], Yusuke Miyazaki[1,2,3], Sari Nurmila[1], Kana Shimizu[1,2], Koji Hasegawa[1,2], Tatsuya Morimoto[1,2,3,*]

[1]Division of Molecular Medicine, School of Pharmaceutical Sciences, University of Shizuoka, Shizuoka, Japan

[2]Division of Translational Research, Clinical Research Institute, Kyoto Medical Center, National Hospital Organization, Kyoto, Japan

[3]Shizuoka General Hospital, Shizuoka, Japan

*Corresponding author:

Tatsuya Morimoto, M.D., Ph.D.

Tel: +81-54-264-5763, Fax: +81-54-264-5744

E-mail: morimoto@u-shizuoka-ken.ac.jp

Keywords: GATA4, Multimerization, Acetylation, Cardiomyocyte, Hypertrophy

Supplementary figure and figure legend

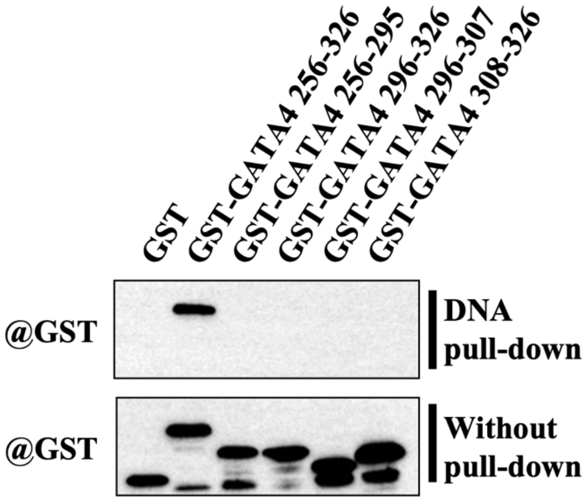

figure S1. Both GATA4 C-terminal zinc finger domain and GATA4 multimerization region were required for DNA binding of GATA4

A biotin-labeled ET-1 probe, which include GATA4 binding sequence, was mixed with GST alone or GST-GATA4 mutants, and then bound to streptavidin beads. The DNA-bound GST fusion GATA4 mutants were detected by Western blotting using anti-GST antibody. The arrow indicates GST alone or GST-GATA4 mutants.

Supplementary method

DNA binding assay

The GST fusion proteins were expressed in *E. coli* BL21 DE3 and mixed with a biotin-labelled ET-1 probe (sense, 5′-BioCCTCTAGAGCCGGGTCTTATCTCCGGCTGCACGTTGC-3′; anti-sense, 5′-GCAACGTGCAGCCGGAGATAAGACCCGGCTCTAGAGG-3′) in DNA binding buffer (0.3 mg/ml bovine serum albumin (BSA), 0.1 mg/ml salmon sperm, 20 mM Hepes pH 7.9, 1.5 mM $MgCl_2$, 400 mM NaCl, 0.2 mM EDTA, 25% glycerol, 0.02% Tween20)

and incubated for 2 h at 4 °C. Streptavidin Sepharose High Performance Beads were added to the protein-DNA mixtures and incubated for 2 h at 4℃ . The beads were then washed four times with wash buffer (20 mM Tris pH 8.0, 2.5 mM $MgCl_2$, 100 mM KCl, 5% glycerol, and 0.1% Tween20), resuspended in 0.1 M glycine (pH 2.5 at 4 °C for 5 min), and subsequently analyzed by Western blotting using anti-GST antibody (Medical & Biological Laboratories, Tokyo, Japan).
